# Online-to-offline combined with problem-based learning is an effective teaching modality in the standardized residency training of nephrology

**DOI:** 10.1186/s12909-024-05675-w

**Published:** 2024-07-02

**Authors:** Junxia Wu, You Ke, Zhida Chen, Mhd Alaa Alhendi, Lina Zhu, Kunling Ma

**Affiliations:** 1 Department of Nephrology, The Second Affiliated Hospital, School of Medicine, Zhejiang University, Hangzhou, 310009 Zhejiang, PR China; 2 School of Medicine, Zhejiang University, Hangzhou, 310009 Zhejiang China

**Keywords:** Online-to-offline, Problem-based learning, Nephrology, Medical education, Residency training in internal medicine

## Abstract

**Background:**

The online-to-offline (O2O) teaching method is recognized as a new educational model that integrates network learning into offline classroom education, while problem-based learning (PBL) is a teaching modality that guides students to apply acquired theoretical knowledge to solve practical problems. However, implementing O2O combined with PBL has not been extensively explored in nephrology residency training. This study aims to explore the efficacy of O2O combined with PBL in the standardized residency training of nephrology by comparing it with the traditional lecture-based teaching (LBT).

**Methods:**

Sixty residency trainees who participated in the standardized training of internal medicine in the nephrology department of the Second Affiliated Hospital of Zhejiang University School of Medicine were equally allocated into O2O combined with PBL (O2O/PBL) or the LBT group demographically matched. Examinations of theory, practice skills, clinical thinking and teaching satisfaction surveys were utilized to assess the teaching effects of the two groups.

**Results:**

Participants from the O2O/PBL group outperformed those from the LBT group in the examination of theory (81.233 ± 9.156 vs. 75.800 ± 7.009, mean ± SEM), practice skills (104.433 ± 3.569 vs.100.316 ± 4.628, mean ± SEM) and clinical thinking (88.933 ± 4.473 vs. 86.667 ± 3.844, mean ± SEM). There was no significant difference in the teaching satisfaction between the two groups.

**Conclusion:**

The current study shows the positive impact of O2O combined with PBL approach on standardized residency training in nephrology without reducing teaching satisfaction.

**Supplementary Information:**

The online version contains supplementary material available at 10.1186/s12909-024-05675-w.

## Background

China has established a system of national standardized medical residency training since 2014, affecting the health of its 1.4 billion people [1]. The Chinese government stated that by 2020, all new medical graduates must first complete residency training in an accredited program before working as attending physicians [2]. Residents are required to finish 33 months of training in different departments within internal medicine in order to complete their training. Moreover, 2 months are arranged for the nephrology department rotations according to the new standards for the internal medicine curriculum [1]. However, nephrology training has been facing a challenging phase, with difficulty in attracting prospective residents. Factors such as lower income, greater complexity compared to other specialties, and relatively limited job opportunities in some parts of the country may all be significant contributing factors. Therefore, improving teaching methods could potentially render nephrology easier to understand and could hopefully enhance recruitment to the field [3]. Thus, it is very important to develop new teaching methods to improve the teaching efficiency of nephrology in the standardized residency training.

The advent of the COVID-19 pandemic has necessitated a transition from the offline face-to-face teaching model to the online model as a measure to mitigate the congregation and subsequent transmission of the virus. Due to its efficiency and immediacy, online live teaching is highly regarded by a substantial number of educators [4]. Ding Talk serves as critical software extensively utilized for office work and online teaching, particularly during the COVID-19 pandemic in China [5]. The online-to-offline (O2O) teaching method is emerging as an innovative educational model that integrates network learning into offline classroom education in the post-epidemic era [6], which has been applied to advanced mathematics education and yields exceptional outcomes [4].

Problem-based learning (PBL) is recognized as an effective, active learning strategy that offers various benefits to students [7, 8]. It is characterized as an innovative teaching method that is problem-based, student-centered, and teacher-guided, which directs students to apply their acquired theoretical knowledge to **resolve** practical challenges [9–14]. This approach has been successfully implemented in both the standardization training for ultrasonography residents as well as in undergraduate medical education [15, 16]. However, the integration of online-to-offline combined with problem-based learning has not been well explored in the education of nephrology in the standardized residency training. Thus, the aim of this study is to explore the effect of O2O combined with the PBL teaching method in the standardized residency training of nephrology.

## Methods

### Participants

A total of 60 internal medicine trainees, ranging in age from 23 to 30 years, were voluntarily enrolled from the nephrology department of the Second Affiliated Hospital of Zhejiang University School of Medicine. These trainees underwent a standardized internal medicine training program in the nephrology department, which spanned from August 1, 2022, to April 30, 2023.

### Study design

Following informed consent, participants were divided into two distinct groups: the traditional lecture-based teaching (LBT) group and the O2O combined with PBL (O2O/PBL) teaching group. The LBT group comprised 30 trainees, including 10 males and 20 females. And the O2O/PBL group comprised 30 trainees, including 8 males and 22 females (Table 1).

**Table 1:** Demographic information of participants in the study

Number of participants (percentage)			
**Gender**	**O2O/PBL(** ***n*** ** = 30)**	**LBT(** ***n*** ** = 30)**	
Male	8(26.67%)	10(33.33%)	
Female	22(73.33%)	20(66.67%)	

O2O/PBL: Online-to-offline combined with problem-based learning; LBT: lecture-based teaching

**Fig. 1:**
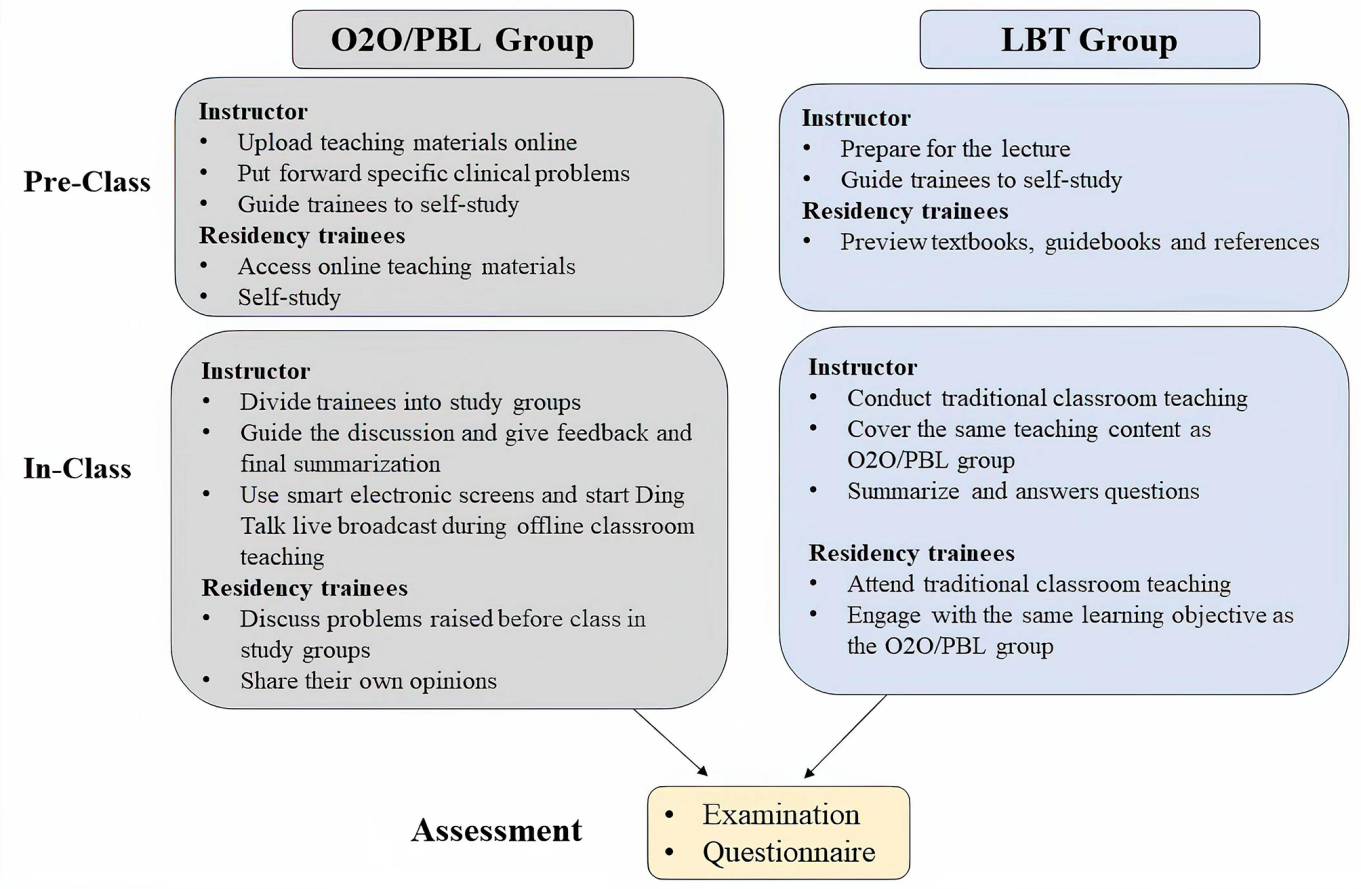
Schematic demonstration of the process of teaching activities. In the traditional LBT model, residency trainees were exposed to new materials in class through lecture delivered by the instructor in the classroom. While residency trainees in O2O/PBL model were exposed to the material prior to class and involved in problem-based learning mode with study groups during the class via face-to-face teaching by Ding Talk broadcast. Live playback of the classes was available after class for repeated viewing to reinforce learning outcomes. O2O/PBL: Online-to-offline combined with problem-based learning; LBT: lecture-based teaching

The training instructors of the two groups are all attending nephrologists who have worked for at least 3 years in the nephrology department of the Second Affiliated Hospital of Zhejiang University School of Medicine. And they are regularly trained by educators who are proficient in O2O/PBL and the LBT teaching methods and have passed regular assessments according to the new standards for residency training of internal medicine. Both groups of residency trainees participated in the teaching activities of the nephrology department, such as residency training lectures, case discussions, and teaching rounds. The teaching content was formulated according to the residency training teaching syllabus in China. The study was carried out following the flowchart as demonstrated in Fig. 1.

The teaching process of O2O/PBL group involved the following: Uploading pre-class materials: including online teaching materials such as operation and theoretical courses, putting forward specific clinical problems, and guiding trainees to self-study before class. The theoretical courses referred to the related cases and summary of knowledge points of the classes in the format of slideshows. Each trainee was expected to take about 30 min to preview these materials, and the amount of online work was comparable to the time needed to review course material with the LBT group. The operation courses referred to the practice skills videos and instructions on how to perform urinary catheterization and physical checkups in nephrology. While for the LBT group, urinary catheterization was taught by bedside demonstration. They were also encouraged to self-study before the class and practice after class but with no problem-based discussion and teaching videos. In the LBT group there were no additional learning materials before the class.

During the course, trainees were divided into study groups by the instructor, each consisting of 2 to 3 trainees. They discussed the problems raised before the class, shared their own opinions, and the teacher gave feedback and summarized the questions. In each class, smart electronic screens were employed as teaching media in the classroom, and Ding Talk live broadcast started during offline classroom teaching. After the course, playback of the live broadcast of the classes was available for repeated watching to reinforce learning outcomes. The teaching process of the LBT group was as follows: trainees were encouraged to preview relevant textbooks, guidebooks and references before class, and the instructors carried out traditional classroom teaching according to the teaching syllabus. The knowledge points covered in the LBT group addressed the same teaching content as those covered in the O2O/PBL group. Furthermore, the stated learning objective for the LBT group was identical to that of the O2O/PBL group. Finally, the instructors were led to summarize and answer questions. During the class, there were no Ding Talk live broadcasts. The students were allowed to ask questions during the lectures, but no problems were proposed during the LBT classes. Both groups of trainees participated in ward work normally, including receiving inpatients, completing physical examinations, writing medical records, and observing clinical operations according to the outline of the standardized training of internal medicine.

### Assessment system

After the rotation of the nephrology department, trainees of both groups participated in examinations, which included theoretical knowledge, practical skills and clinical thinking, according to the outline of residency training of nephrology. The examination results were employed as objective evaluation indicators. The full score of the theory and clinical thinking examination is 100 points, while the practical skills exam has a full score of 110 points. The theoretical examinations were formulated by the residency training secretary of the nephrology department and underwent a rigorous review by internal medicine educators (Additional file 1). The practical skills examination focused on the proficiency in urinary catheterization. During the clinical thinking examination, trainees were tasked with randomly selected case studies which they analyzed to summarize their medical histories, to give diagnosis, and the differential diagnosis, as well as to propose treatment strategies and interventions. An examiner will score the candidates’ professional knowledge, humanistic, professional quality, and communication ability. The entire examination process was supervised by the residency training secretary of the nephrology department and the supervisory committee of the medical residency training program, ensuring adherence to the established standards [2, 17].

All residency trainees who participated in the study were invited to complete an anonymous survey online (Additional file 2) [18]. The trainees from both the traditional LBT group and O2O/PBL group were respectively assessed via questionnaires to evaluate their subjective perceptions of different teaching methods. The questionnaire covered five aspects: overall teaching satisfaction, learning difficulty, learning interest, classroom atmosphere and knowledge points consolidation. The questionnaire was assessed using a five-point scale, where a score of 1 indicated “very satisfied” and a score of 5 denoted “very dissatisfied”, with intermediate values representing “relatively satisfied”, “generally satisfied”, and “not very satisfied” [19, 20].

### Data evaluation and statistical analysis

SPSS 22.0 was utilized for statistical analysis of the theoretical scores, practical skills scores, clinical thinking scores, and teaching satisfaction survey items. All measurement data were represented by (mean ± standard deviation). Normal tests were conducted on the data, and the test scores were in line with the normal distribution, whereas the teaching satisfaction survey items were not in line with the normal distribution. An independent sample t-test [21] was applied for test scores and a chi-square test was utilized for comparison of teaching satisfaction between the two groups. *P* < 0.05 was considered as statistically significant, *P*>0.05 was considered as not significant. All analytical methods were conducted in accordance with relevant guidelines and regulations.

## Results

### Comparative analysis of the test score

The theoretical score of O2O/PBL group was 81.233 ± 9.156, and that of the LBT group was 75.800 ± 7.009. The practical skills score of O2O/PBL group was 104.433 ± 3.569, and that of the LBT group was 100.316 ± 4.628. The clinical thinking score of the O2O/PBL group was 88.933 ± 4.473, and that of the LBT group was 86.667 ± 3.844. The differences were all statistically significant (*P* < 0.05), as shown in Table 2. We also performed an ANOVA test and obtained similar results to the independent sample t-test for the three test scores between the two groups (Additional file 3).

**Table 2:** Comparison of theoretical assessment, practical skills and clinical thinking scores between the two groups (mean ± standard deviation)

group	theoretical assessment	practical skills	clinical thinking
O2O/PBL	81.233 ± 9.156	104.433 ± 3.569	88.933 ± 4.473
LBT	75.800 ± 7.009	100.316 ± 4.628	86.667 ± 3.844
t	2.581	2.825	2.105
*P*	0.013	0.006	0.04

Independent sample t-test was adopted. *P* < 0.05 was considered as statistically significant. O2O/PBL: Online-to-offline combined with problem-based learning; LBT: lecture-based teaching

### Comparative analysis of questionnaire results

**Fig. 2:**
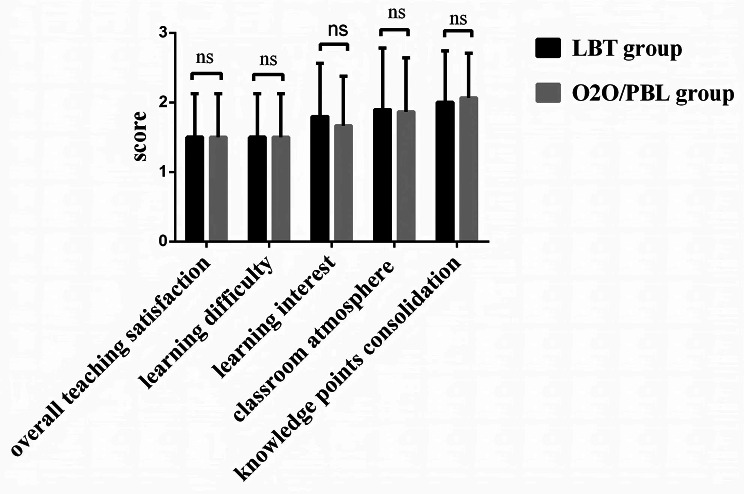
Comparison of teaching satisfaction evaluation. A Chi-square test was employed for comparison between groups, *P*>0.05 was considered as not significant. NS: not significant

In this study, a total of 60 questionnaires were distributed and all 60 were received. The questionnaire survey revealed that in the O2O/PBL group, 17 people were very satisfied, 11 people were relatively satisfied, 2 people were generally satisfied, and the satisfaction rate was 93.33%; in the LBT group, 17 people were very satisfied, 11 people were relatively satisfied, 2 people were generally satisfied, and the overall satisfaction rate was 93.33%. Teaching satisfaction survey showed no statistical difference in overall teaching satisfaction, learning difficulty, learning interest, classroom atmosphere, and knowledge points consolidation, as shown in Fig. 2 (*P* > 0.05).

## Discussion

In the post-epidemic era, the **single** online or offline teaching method cannot meet the needs of medical education. The teaching platform is very important for developing online teaching activities as it serves as a bridge connecting teachers and students while carrying various teaching resources [6, 22]. A smart screen with access online was provided by the Second Affiliated Hospital of Zhejiang University School of Medicine for teaching, meeting, and remote consultation. Ding Talk and smart screens make it convenient to start a live stream while conducting face-to-face teaching in the classroom. Live playback of the classes was available after class for repeated viewing to reinforce learning outcomes.

Nephrology is a comprehensive and highly practical discipline which is an important part of standardized residency training in internal medicine. **Nephrology education** is a demanding, complex and often frustrating task. Therefore, the implementation of innovative teaching methods is crucial for enhancing teaching quality and **strengthening** students’ learning initiative and enthusiasm [21, 23–26].

The O2O teaching method refers to an innovative teaching method that integrates online with offline teaching, which both have the advantage of the ease and efficiency of online education and the environmental experience of offline education. It has been proven that the O2O teaching method has relatively powerful and outstanding practical and social characteristics [27, 28]. PBL teaching method is student-centered, combined with specific cases [10]. It also stimulates students’ subjective initiative in learning through pre-class learning, in class group discussion, and self-study after class. The PBL approach has gained substantial traction in the realm of clinical education due to its effectiveness in enhancing clinical thinking and practical skills [29, 30].

Based on these theories and experiences, we combined O2O and PBL teaching methods into the residency training of internal medicine of nephrology, utilizing smart electronic screen as the teaching medium, distributing pre-class and after-class learning materials through Ding Talk network platform, assigning pre-class and after-class homework, initiating Ding Talk live broadcast during face-to-face teaching session, and facilitating students’ discussions on clinical problems raised by instructors in study groups. The teacher summarized the questions, gave feedback and answered questions raised by the students. Finally, a statistical analysis was conducted on the students’ theoretical scores, practical skills scores, and clinical thinking assessment scores (Fig. 1). We found that the test scores of the O2O combined PBL teaching group were higher than those of the traditional teaching group, and the difference was statistically significant (Table 2). Furthermore, the teaching satisfaction of two groups of students was investigated. There was no statistically significant difference between the two groups, and the complaint rate did not increase (Fig. 2). These findings suggest that the O2O combined PBL teaching method was more effective than the traditional lecture-based teaching because it can significantly improve the learning effect of residency trainees without reducing teaching satisfaction. This study showed that the O2O combined PBL teaching method is promising and could be promoted in the standardized residency training of nephrology.

There are two limitations to our study. One is that we conducted the study only in our institution with a relatively small number of learners. Another limitation is that we assessed only the short-term results of this teaching approach via test scores. But our research provides a foundation for the O2O combined PBL teaching method to be conducted as an effective teaching method and applied in other institutions with more learners. Further studies are needed to investigate the long-term benefits of this teaching approach for internal medicine training in nephrology such as educational policy making and even career development in China and other countries in the future.

## Conclusion

In summary, by comparing the learning effect of O2O/PBL and LBT in residency training of nephrology, we believe that O2O/PBL teaching method can provide a better teaching method for residency training of nephrology, which can fully mobilize students’ learning initiative and achieve better learning results.

### Electronic supplementary material

Below is the link to the electronic supplementary material.

Supplementary Material 1

Supplementary Material 2

Supplementary Material 3

## Data Availability

The related materials including teaching materials, examination materials, and teaching satisfaction surveys were kept in hard copies in the nephrology department of the Second Affiliated Hospital of Zhejiang University, School of Medicine. The original datasets of the study are available from the corresponding author on reasonable request.

## Abbreviations

O2O/PBL: Online-to-offline combined with problem-based learning
LBT: lecture-based teaching
