# Supplementary material for: Online-to-offline combined with problem-based learning is an effective teaching modality in the standardized residency training of nephrology
Source: BMC Med Educ. 2024 Jul 2;24:712. doi: 10.1186/s12909-024-05675-w (PMC11221083; doi:10.1186/s12909-024-05675-w)
Supplement: Supplementary file 1 — Supplementary Material 1 [file 12909_2024_5675_MOESM1_ESM.docx]

**Supplementary information**

Additional file 1 of Online-to-offline combined with problem-based learning is an effective teaching modality in the standardized residency training of nephrology

The content of theory examination and survey were provided in additional file 1.

Additional file 1

Supplementary Quiz

1. The highly specific renal pathological changes in diabetic nephropathy are (2 points)

A. Diffuse glomerulosclerosis

B. Nodular glomerulosclerosis

C. Exudative lesions

D. Focal segmental glomerulosclerosis

E. Crescentic glomerulonephritis

2. Chronic kidney failure can lead to renal osteodystrophy due to the lack of hormones secreted by the kidneys (2 points)

A. Erythropoietin

B. Renin

C. Calcitriol

D. Prostaglandin

E. Parathyroid hormone

3. The renal vascular disease that does not cause acute renal failure is (2 points)

A. Scleroderma renal crisis

B. Cholesterol embolism in renal arterioles

C. Benign arteriosclerosis of renal arterioles

D. Malignant hypertension

E. Hemolytic uremic syndrome

4. The relative contraindications for hemodialysis include all except (2 points)

A. Psychiatric disorder patients

B. Patients with shock or hypotension (systolic blood pressure < 80mmHg)

C. Severe bleeding tendency or cerebral hemorrhage

D. Severe hypertension

E. Pulmonary edema and heart failure caused by severe myocardial disease

5. The most commonly used laboratory test to assess glomerular filtration function is (2 points)

A. Inulin clearance rate

B. Creatinine clearance rate

C. Urine specific gravity

D. Urine concentration and dilution test

E. Urine osmolality

6. Long-term peritoneal dialysis treatment in uremic patients should be discontinued if (2 points)

A. Severe and persistent vomiting

B. Pulmonary infection

C. Drainage obstruction

D. Abdominal pain and diarrhea

E. Improvement in kidney function

7. The following are all causes of prerenal renal insufficiency except (2 points)

A. Hemorrhage

B. Malignant hypertension

C. Excessive diuresis

D. Severe diarrhea

E. Cardiac tamponade

8. Which statement about electrolyte imbalance in chronic renal failure patients is incorrect? (2 points)

A. When the glomerular filtration rate drops to 20-25ml/min or lower, the kidney's potassium excretion capacity gradually declines, and hyperkalemia is likely to occur.

B. Most patients with early-stage renal failure have hyperphosphatemia.

C. Hypocalcemia and hyperphosphatemia can only be detected in the middle and late stages of renal failure, and usually do not cause clinical symptoms.

D. When GFR < 20ml/min, reduced renal magnesium excretion can lead to mild hypermagnesemia.

E. Elevated blood phosphorus can inhibit the proximal tubule from producing calcitriol, which is important for maintaining normal blood calcium levels, and its deficiency can lead to lower blood calcium levels.

9. Thrombotic complications in nephrotic syndrome patients are most common in the (2 points)

A. Renal vein

B. Coronary vessels

C. Lower limb veins

D. Inferior vena cava

E. Pulmonary vein

10. A 68-year-old female patient was found to have multiple cysts of varying sizes in the left renal cortex during an ultrasound examination. (2 points)

A. Solitary renal cyst

B. Multiple renal cysts

C. Polycystic kidney

D. Renal pelvic cyst

E. Medullary sponge kidney

11. Adverse reactions to cyclosporine treatment for nephrotic syndrome do not include (2 points)

A. Hepatotoxicity

B. Nephrotoxicity

C. Hypertension

D. Hirsutism

E. Hair loss

12. The most significant diagnostic feature for acute glomerulonephritis is (2 points)

A. Proteinuria and hyaline casts

B. Proteinuria and pyuria

C. Hematuria and leukocytes

D. Hematuria and red blood cell casts

E. Large numbers of leukocytes and leukocyte casts in the urine

13. The incorrect clinical classification of primary glomerular disease is (2 points)

A. Acute glomerulonephritis

B. Rapidly progressive glomerulonephritis

C. Nephrotic syndrome

D. Chronic glomerulonephritis

E. IgA nephropathy

14. The incorrect option regarding the clinical classification of primary glomerular disease is (2 points)

A. Acute glomerulonephritis

B. Rapidly progressive glomerulonephritis

C. Chronic glomerulonephritis

D. Sclerosing glomerulonephritis

E. Nephrotic syndrome

15. The laboratory characteristics consistent with type I renal tubular acidosis are (2 points)

A. Hyperchloremic metabolic acidosis

B. Hyperkalemia

C. Hypercalcemia

D. Urine pH < 5.5

E. Hypernatremia

16. Incorrect statement regarding the body's defense against urinary tract infections (2 points)

A. Urine flushes away most bacteria

B. Hamm-Horsfall protein in urine promotes bacterial adhesion to the urinary epithelium

C. Immunoglobulins in the urine can kill bacteria

D. Mucopolysaccharides on the bladder surface prevent bacterial adhesion

E. Low urine pH inhibits bacterial growth

17. Incorrect description of chronic pyelonephritis treatment (2 points)

A. Remove the predisposing factors

B. Use effective drugs alone for 2-4 weeks

C. For mild symptoms, low-dose antibacterial therapy can be used

D. Treat acute episodes similarly to acute pyelonephritis

E. Choose antibiotics based on urine culture and sensitivity

18. The typical duration of antibiotic treatment for acute cystitis in pregnant women is (2 points)

A. 1 day

B. 3 days

C. 1 week

D. 2 weeks

E. 6 weeks

19. Incorrect statement regarding indications for dialysis in chronic renal failure (2 points)

A. Dialysis is needed only when blood creatinine levels exceed 707 μmol/L

B. Emergency dialysis should be arranged for signs of pulmonary or cerebral edema

C. Emergency dialysis should be arranged when serum potassium levels exceed 6.5 mmol/L

D. Dialysis should be initiated when GFR is 6-10 ml/min with significant uremic symptoms that do not improve with treatment

E. For diabetic nephropathy, dialysis should be scheduled earlier (GFR 10-15 ml/min)

20. Adverse reactions to cyclophosphamide treatment for nephrotic syndrome do not include (2 points)

A. Bone marrow suppression

B. Toxic liver damage

C. Gonadal suppression

D. Hair loss

E. Renal impairment

21. Which blood purification method should be avoided for most patients after extensive mesenteric resection? (2 points)

A. Intermittent hemodialysis

B. CRRT

C. Peritoneal dialysis

D. Hemoperfusion

E. Plasma exchange

22. Incorrect management for preventing acute renal failure in nephrotic syndrome is (2 points)

A. Loop diuretics can be used

B. Hemodialysis is used if diuretics are ineffective

C. Treat the underlying disease actively

D. Strong diuretics

E. Alkalinize the urine

23. Most suitable candidate for peritoneal dialysis is (2 points)

A. Diabetic patients with renal failure

B. Uremic patients with stable cardiovascular function

C. Polycystic kidney patients with renal failure

D. Renal failure patients with good lung function

E. Patients with multiple myeloma and renal failure

24. The main mechanism of edema caused by acute glomerulonephritis is (2 points)

A. Increased capillary permeability

B. Secondary heart failure

C. Secondary hyperaldosteronism

D. Hypoproteinemia and decreased plasma colloid osmotic pressure

E. Decreased glomerular filtration rate, water, and sodium retention

25. Granular casts can be seen in the following diseases, except (2 points)

A. Chronic nephritis

B. Pyelonephritis

C. Late-stage acute glomerulonephritis

D. Drug-induced renal tubular damage

E. Acute glomerulonephritis

26. Incorrect description of multiple myeloma is (2 points)

A. Acute renal failure is the leading cause of death

B. Amyloidosis can occur

C. Excessive uric acid can deposit in renal tubules, leading to uric acid nephropathy

D. Serum alkaline phosphatase is usually normal or slightly elevated

E. Bone pain is often the main early symptom

27. Incorrect statement about the pathogenesis of glomerular disease (2 points)

A. It is an immune-mediated inflammatory disease

B. Immune mechanisms are the primary cause

C. The immune response includes cellular and humoral immunity

D. Non-immune mechanisms play no role

E. Inflammatory mediators include complement, interleukins, reactive oxygen species, etc.

28. To increase the ultrafiltration volume of peritoneal dialysis, the following methods are commonly used (2 points)

A. Prolong the retention time of dialysis solution in the abdominal cavity

B. Add a suitable amount of albumin to the dialysis solution

C. Increase the glucose concentration in the dialysis solution

D. Use a peritoneal dialysis machine

E. Increase the frequency of dialysis solution exchanges

29. Complement level reduction in the blood is generally not seen in (2 points)

A. Rapidly progressive glomerulonephritis

B. Membranous nephritis

C. Mesangial proliferative glomerulonephritis

D. Lupus nephritis

E. Acute glomerulonephritis

30. The main reason for hypertension in most chronic renal failure patients is (2 points)

A. Increased plasma renin activity

B. Water and sodium retention, excessive volume load

C. Reduced prostaglandin secretion

D. Reduced secretion of vasorelaxants

E. Excessive catecholamine secretion

31. The incorrect statement about acute glomerulonephritis is (2 points)

A. Treatment is mainly rest and symptomatic management

B. This disease is self-limiting

C. Glucocorticoids and cytotoxic drugs are used

D. Dialysis should be provided to patients with acute renal failure

E. Rest in bed during the acute phase until gross hematuria disappears

32. A 25-year-old male patient had microscopic hematuria after an upper respiratory tract infection six months ago. Physical examination: BP 150/120 mmHg. Urinalysis showed proteinuria (++), red blood cells 5-10/HP. Normal renal function. Renal biopsy and histopathology showed IgA nephropathy with partial glomerulosclerosis and renal tubular atrophy. Which of the following factors is least related to prognosis? (2 points)

A. Degree of hypertension

B. Speed of renal function deterioration

C. Degree of hematuria

D. Degree of glomerulosclerosis

E. Amount of proteinuria

33. A 70-year-old male patient had coronary artery disease for more than 10 years. Two days after coronary angiography, he developed reduced appetite, nausea, and edema, with blood pressure 140/90 mmHg, pulse 78 bpm, creatinine 231 μmol/L, and hemoglobin 120 g/L. The most likely diagnosis is (2 points)

A. Acute tubular necrosis

B. Chronic renal insufficiency

C. Rapidly progressive glomerulonephritis

D. Prerenal acute renal insufficiency

E. Acute interstitial nephritis

34. A 40-year-old male patient had a 10-year history of peptic ulcer. In the last two days, his abdominal pain worsened, he had recurrent vomiting, couldn't eat or drink water, and his urine output reduced yesterday to about 150-350 ml/day. Laboratory tests: SCr 556 μmol/L, urine specific gravity 1.022, and proteinuria (±). The most likely diagnosis is (2 points)

A. Acute glomerulonephritis

B. Rapidly progressive glomerulonephritis

C. Acute tubular necrosis

D. Prerenal acute kidney injury

E. Chronic renal failure

35. A 33-year-old male patient diagnosed with chronic glomerulonephritis. Physical examination: blood pressure 135/80 mmHg, no edema, 24-hour urinary protein quantification 0.3-0.7 g/day, serum creatinine 116 μmol/L. The best drug treatment is (2 points)

A. Low molecular weight heparin

B. Angiotensin-converting enzyme inhibitors

C. Calcium channel blockers

D. Glucocorticoids

E. Cytotoxic drugs

36. A 28-year-old female patient had recurrent gross hematuria for more than 2 years. Five days after a respiratory infection, she had gross hematuria again. Physical examination: mild swelling of both lower limbs, blood pressure 120/80 mmHg. Urinalysis showed protein (++), red blood cells (++++). To confirm the diagnosis, the most significant test is (2 points)

A. Renal ultrasound

B. Renal biopsy

C. Renal CT

D. Intravenous pyelography

E. Midstream urine culture

37. A female patient had recurrent urinary frequency, urgency, and hematuria, and urine culture was positive for Mycobacterium tuberculosis. CT revealed pyonephrosis in the right kidney. The best treatment is (2 points)

A. Anti-infective treatment

B. Anti-tuberculosis treatment

C. Anti-tuberculosis + lesion removal

D. Anti-tuberculosis + right nephrectomy

E. Anti-tuberculosis + partial nephrectomy

38. A 14-year-old boy came to the hospital due to abdominal pain. Physical examination: symmetrical, patchy petechiae on both lower limbs. Urinalysis: Pro (-), RBC 20/HP, WBC 1-2/HP. The most likely diagnosis is (2 points)

A. Renal vascular malformation

B. Henoch-Schönlein purpura nephritis

C. Renal colic

D. Acute pyelonephritis

E. Systemic lupus erythematosus

39. A 40-year-old male patient had intermittent edema for 10 years, nausea and vomiting for 1 week. Blood pressure 20/13.3 kPa (150/100 mmHg), Hb 80 g/L, proteinuria (++), waxy casts (+), blood BUN 40 mmol/L, SCr 760 μmol/L, serum potassium 5.5 mmol/L. The most appropriate initial treatment is (2 points)

A. Antihypertensive treatment

B. Diuretics

C. Correct anemia

D. Dietary treatment

E. Hemodialysis

40. A 15-year-old male patient had oliguria and edema for 5 days, cough, and shortness of breath, unable to lie flat for 1 day. Two weeks before the onset, he had a sore throat for 3 days. BP 170/110 mmHg, sitting breathing, scattered wet rales at the lung bases, urine specific gravity 1.022, proteinuria (+++), red blood cells 30-90/HP, and low C3 complement level. The diagnosis is (2 points)

A. Acute nephritis with left heart failure

B. Acute exacerbation of chronic nephritis

C. Hypertension with left heart failure

D. Rapidly progressive nephritis with left heart failure

E. Nephrotic syndrome with left heart failure

41. An 18-year-old female patient had sore throat, cough, headache, and fatigue for 2 weeks, then developed turbid reddish-brown urine, without urinary frequency, urgency, or pain, facial edema, no fever or chills. BP 150/90 mmHg, Hb 102 g/L, ESR 45 mm/h, mildly elevated serum creatinine and urea nitrogen, significantly elevated antistreptolysin O, low C3 complement. The diagnosis is acute glomerulonephritis. The turbid reddish-brown urine is (2 points)

A. Myoglobinuria

B. Hemoglobinuria

C. Gross hematuria

D. Hematoporphyrinuria

E. Chyluria

42. A 35-year-old male patient developed a rash, itching, and low fever all over the body after taking allopurinol for 3 weeks. Urinalysis showed leukocytes (+), proteinuria (+), specific gravity 1.010, serum creatinine 192 μmol/L, blood glucose 4.2 mmol/L. The most likely diagnosis is (2 points)

A. Acute drug-induced allergic interstitial nephritis

B. Chronic interstitial nephritis

C. Diabetic nephropathy

D. Chronic glomerulonephritis

E. Acute glomerulonephritis

43. Which of the following is not a basis for diagnosing this case? (2 points)

A. Recent medication history

B. Rash with itching

C. Abnormal urine test

D. Elevated blood glucose

E. Damage to renal tubule and glomerular function

44. The incorrect treatment plan for this case is (2 points)

A. Discontinue medication

B. Symptomatic treatment

C. Glucocorticoids can be used

D. Dialysis should be given if acute renal failure occurs

E. Cytotoxic drugs should be used

45. A 56-year-old male patient presented to the outpatient department with frequent urination, urgency, dysuria, and gross hematuria for 3 months. Physical examination: good general condition, mild anemia, no palpable kidneys, clear percussion in the bladder area. The primary clinical diagnosis of this case should be (2 points)

A. Urinary tract infection

B. Benign prostatic hyperplasia

C. Urinary tuberculosis

D. Bladder stones

E. Urinary tract tumor, likely bladder tumor

46. If the lesion is in the kidney, the characteristic of hematuria is (2 points)

A. Painless gross hematuria throughout

B. Gross hematuria throughout with bladder irritation

C. Initial hematuria

D. Painful hematuria

E. Hemoglobinuria

47. A 30-year-old female patient had a regular health check-up in the past, with negative urine protein and normal renal function. After fever 1 week ago, she developed nausea and vomiting, and her serum creatinine was 340 μmol/L. After admission, a renal biopsy was performed, and pathology showed crescents in 60% of the glomeruli. The clinical diagnosis was rapidly progressive glomerulonephritis, and the pathological diagnosis was type I crescentic nephritis. The treatment should include (2 points)

A. Adrenal cortical hormones, indomethacin

B. Adrenal cortical hormone pulse therapy, peritoneal dialysis

C. Adrenal cortical hormone pulse therapy, hemodialysis

D. Adrenal cortical hormone pulse therapy, plasma exchange

E. Adrenal cortical hormone pulse therapy, kidney transplant

48. The main factors for judging the prognosis of this disease do not include (2 points)

A. Timing of intensive treatment

B. Age

C. Proteinuria

D. Percentage of glomerular crescents in the renal biopsy

E. Renal function

49. A 28-year-old male patient had recurrent eye edema for 10 years, recently developed fatigue, pale skin, oliguria, BP 160/100 mmHg, difficulty breathing, small bubbling sounds at the lung bases, and couldn't lie flat. BUN 30 mmol/L, serum creatinine 1001 μmol/L. Diagnosed with uremia. The main treatment currently should be (2 points)

A. Antihypertensive medication

B. Furosemide 100 mg, twice daily, IV injection

C. Proscillaridin 0.2 mg, once daily, IV injection

D. Vasodilators and proscillaridin treatment

E. 5% NAHCO3 60 ml, once daily, IV injection

50. When the creatinine clearance rate is below 10 ml/min in uremic patients, with significant left heart failure, the best treatment should be (2 points)

A. Diuretics

B. Cardiotonic drugs

C. Vasodilators

D. Antihypertensive drugs

E. Dialysis therapy
