# Supplementary material for: Online-to-offline combined with problem-based learning is an effective teaching modality in the standardized residency training of nephrology
Source: BMC Med Educ. 2024 Jul 2;24:712. doi: 10.1186/s12909-024-05675-w (PMC11221083; doi:10.1186/s12909-024-05675-w)
Supplement: Supplementary file 2 — Supplementary Material 2 [file 12909_2024_5675_MOESM2_ESM.docx]

**Supplementary information**

Additional file 2 of Online-to-offline combined with problem-based learning is an effective teaching modality in the standardized residency training of nephrology

Additional file 2

Supplementary Survey

**Survey**

1. How satisfied are you with the overall teaching of nephrology? ( )

Options:

A. Very satisfied

B. Relatively satisfied

C. Neutral

D. Not very satisfied

E. Very dissatisfied

2. How difficult do you find nephrology? ( )

A. Very easy

B. Relatively easy

C. Moderate

D. Relatively difficult

E. Very difficult

3. How do you find the classroom atmosphere in nephrology teaching? ( )

A. Very good

B. Relatively good

C. Neutral

D. Not very good

E. Very poor

4. Do you think your interest in learning nephrology has been stimulated? ( )

A. Highly stimulated

B. Fairly stimulated

C. Neutral

D. Not very stimulated

E. Not stimulated at all

5. Do you feel that your knowledge has become more solid through nephrology teaching? ( )

A. Very solid

B. Fairly solid

C. Neutral

D. Not very solid

E. Not solid at all
