# Supplementary material for: Online-to-offline combined with problem-based learning is an effective teaching modality in the standardized residency training of nephrology
Source: BMC Med Educ. 2024 Jul 2;24:712. doi: 10.1186/s12909-024-05675-w (PMC11221083; doi:10.1186/s12909-024-05675-w)
Supplement: Supplementary file 3 — Supplementary Material 3 [file 12909_2024_5675_MOESM3_ESM.docx]

**Supplementary information**

Additional file 3 of Online-to-offline combined with problem-based learning is an effective teaching modality in the standardized residency training of nephrology

Comparison of theoretical assessment, practical skills and clinical thinking scores between the two groups (mean ± standard deviation)

| group | theoretical assessment | practical skills | clinical thinking |
| --- | --- | --- | --- |
| O2O/PBL | 81.233±9.156 | 104.433±3.569 | 88.933±4.473 |
| LBT | 75.800±7.009 | 100.316±4.628 | 86.667±3.844 |
| F | 6.660 | 7.980 | 4.430 |
| P | 0.012 | 0.006 | 0.04 |

An ANOVA test was adopted. P<0.05 was considered as statistically significant. O2O/PBL: Online-to-offline combined with problem-based learning; LBT: lecture-based teaching.
